# 

**Published:** 1879-11-20

**Authors:** 


### *EDITORIAL NOTICES.*

☞ *Mistake.*—An article in our September number attributed to H. W. Carpenter should have been credited to W. W. Carpenter, of California.

H. P. TRUMAN & Co.—See advertisement of this fine establishment of Louisville. Their batteries are excellent, and come highly recommended by leading men in the profession.

MESSRS. REED & CARNRICK, manufacturing pharmacists, New York, have made a very liberal and handsome contribution of their beautiful preparations and chemicals to the Medical College Dispensary, which are acknowledged and cordially appreciated. We invite attention also to an advertisement of this splendid establishment commencing in the present number of our journal.

APPLETON & Co.—This large, enterprising and liberal publishing house has donated to the College Library thirty-six volumes of valuable books. They were opened too late to permit the publication of a list of the works, which we will do in our next issue.

Other valuable donations have been received, but too late for notice in this issue.

RECEIPTED.—Dr. Levi Farrow; Dr. L. C. Harvey, 1879; Dr. J. F. DeLonne, to August, 1880; Dr. J. P. Simons, Dr. W. A. Cusick, W. D. Hunt, R. Imgrus, John B. Foster. 1879; — McCall to July, 1880; T. L. Lallerstedt, 1879; J. D. Jordan, 1879—1880; T. W. Sproull, 1879; W. H. Walton, to October, 1880; E. G. Whitman, 1879; J. F. Fonville, 1880; John T. Booth. 1879; Thos. S. Jones, 1880; Robt. Kelly, 1879.

